# Linked decreases in liver kinase B1 and AMP-activated protein kinase activity modulate matrix catabolic responses to biomechanical injury in chondrocytes

**DOI:** 10.1186/ar4254

**Published:** 2013-07-25

**Authors:** Freyr Petursson, Matt Husa, Ron June, Martin Lotz, Robert Terkeltaub, Ru Liu-Bryan

**Affiliations:** 1 VA San Diego Healthcare System, Department of Medicine, University of California San Diego, 111K, 3350 La Jolla Village Drive, San Diego, CA 92161, USA; 2 Department of Medicine, The Ohio State University, 480 Medical Center Drive, Columbus, OH 43210, USA; 3 Mechanical and Industrial Engineering Department, Montana State University, PO Box 173800 Bozeman, MT 59717-3800, USA; 4 The Scripps Research Institute, 10550 North Torrey Pines Road, La Jolla, CA 92037, USA

**Keywords:** osteoarthritis, cartilage, aging, MMP-3, nitric oxide

## Abstract

**Introduction:**

AMP-activated protein kinase (AMPK) maintains cultured chondrocyte matrix homeostasis in response to inflammatory cytokines. AMPK activity is decreased in human knee osteoarthritis (OA) chondrocytes. Liver kinase B1 (LKB1) is one of the upstream activators of AMPK. Hence, we examined the relationship between LKB1 and AMPK activity in OA and aging cartilages, and in chondrocytes subjected to inflammatory cytokine treatment and biomechanical compression injury, and performed translational studies of AMPK pharmacologic activation.

**Methods:**

We assessed activity (phosphorylation) of LKB1 and AMPKα in mouse knee OA cartilage, in aging mouse cartilage (6 to 24 months), and in chondrocytes after mechanical injury by dynamic compression, via immunohistochemistry or western blot. We knocked down LKB1 by siRNA transfection. Nitric oxide, matrix metalloproteinase (MMP)-3, and MMP-13 release were measured by Griess reaction and ELISA, respectively.

**Results:**

Knockdown of LKB1 attenuated chondrocyte AMPK activity, and increased nitric oxide, MMP-3 and MMP-13 release (*P *<0.05) in response to IL-1β and TNFα. Both LKB1 and AMPK activity were decreased in mouse knee OA and aged knee cartilage, and in bovine chondrocytes after biomechanical injury. Pretreatment of bovine chondrocytes with AMPK activators AICAR and A-769662 inhibited both AMPKα dephosphorylation and catabolic responses after biomechanical injury.

**Conclusion:**

LKB1 is required for chondrocyte AMPK activity, thereby inhibiting matrix catabolic responses to inflammatory cytokines. Concurrent loss of LKB1 and AMPK activity in articular chondrocytes is associated with OA, aging and biomechanical injury. Conversely, pharmacologic AMPK activation attenuates catabolic responses to biomechanical injury, suggesting a potentially novel approach to inhibit OA development and progression.

## Introduction

Osteoarthritis (OA) is a disorder of the synovial joint, which culminates in articular cartilage degeneration, and associated pain and disability [1,2]. Age and biomechanical injury are implicated as primary risk factors for OA [1,2]. Chondrocytes, the sole cells residing within articular cartilage, are responsible for maintaining the homeostatic balance between matrix anabolism and catabolism [1,2]. However, biomechanical injury initiates a sequence of biological events in the joint, in which chondrocyte dysfunction and loss of viability lead to progressive articular cartilage damage [3]. In addition, aged chondrocytes exhibit an impaired ability to respond to mechanical and inflammatory insults, manifested as decreased anabolic activity and an increase in catabolic activity, thereby compromising cartilage extracellular matrix integrity [4,5].

IL-1β, TNFα, and other inflammatory mediators in injured and aging joints [6-9] play a significant role in promoting catabolism of type II collagen and proteoglycans [2]. The serine/threonine protein kinase AMP-activated protein kinase (AMPK), a fuel-sensing, master regulator of energy homeostasis and cellular metabolism [10,11], exerts anti-inflammatory effects, partly mediated by inhibition of NF-κB signaling [12]. We demonstrated that AMPK activity is constitutively present in normal articular chondrocytes, but is decreased in human knee OA chondrocytes [13]. IL-1β and TNFα induce marked loss of AMPK activity in normal articular chondrocytes [13]. Conversely, AMPK pharmacological activators attenuate cartilage explant and monolayer cultured chondrocyte procatabolic responses to IL-1β and TNFα [13]. Hence, decreased AMPK activity in articular chondrocytes has the potential to disrupt cartilage homeostasis by promoting matrix catabolism, thereby contributing to progression of OA.

AMPK activation is induced by several upstream kinases via AMPKα subunit phosphorylation at a conserved threonine; dephosphorylation by protein phosphatases inactivates AMPK [10,11]. Liver kinase B1 (LKB1), a serine/threonine protein kinase that was first identified as a tumor suppressor, is one of the upstream kinases that activate AMPK [10,11]. Here, we determined the relationship between LKB1 and AMPK activities in cultured chondrocytes, and examined phosphorylation of LKB1 and AMPKα in human knee OA chondrocytes, in mouse knee OA and aging cartilages, and in bovine knee chondrocytes embedded in alginate after biomechanical injury. Our results closely link matrix catabolism with decreased LKB1 and AMPK activities, present in OA, aging, and injured chondrocytes. Conversely, we establish that AMPK pharmacologic activators inhibit catabolic responses following biomechanical injury in chondrocytes.

## Materials and methods

### Reagents

All chemical reagents were obtained from Sigma-Aldrich (St Louis, MO, USA), unless otherwise indicated. AMPK pharmacologic activators 5-aminoimidazole-4-carboxyamide ribonucleoside (AICAR) and A-769662 were from Tocris Bioscience (Bristol, UK). Recombinant human IL-1β and TNFα, and matrix metalloproteinase (MMP)-3 and MMP-13 ELISA kits were purchased from R&D Systems, Inc. (Minneapolis, MN, USA). Antibodies to phospho-LKB1 (Ser428), phospho-AMPKα (Thr172), total AMPKα and cleaved caspase-3 were from Cell Signaling Technology, Inc. (Danvers, MA, USA) for western blot, and from Abcam (Cambridge, MA, USA) for immunohistochemistry. Human LKB1 siRNA and control siRNA were from Santa Cruz Biotechnology (Santa Cruz, CA, USA).

### Studies of human knee articular chondrocytes

Studies were performed in compliance with an institutionally reviewed and approved human subject protocol by the IRB at the Scripps Research Institute (La Jolla, CA, USA). The human knee chondrocytes were isolated from autopsy donors (no consent was needed) that were graded macroscopically according to a modified Outerbridge scale [14,15]. Grade I represents intact cartilage surface (normal); grade II represents minimal fibrillation (OA); and grade III represents overt fibrillation (OA) [14,15]. Human chondrocytes were cultured in high-glucose DMEM with 10% FCS, 100 μg/ml streptomycin, and 100 IU/ml penicillin at 37°C, and no later than first passage chondrocytes were used for all experiments. Unless otherwise indicated, chondrocytes were plated at 2.5 × 10^5 ^cells per well in 250 μl medium on the day before the experimental treatment in 12-well plates.

### Experimental osteoarthritis models in mice

All mouse experiments were performed in compliance with an institutionally reviewed and approved protocol by IACUC at the Scripps Research Institute. Joint instability-induced OA was induced in 2-month-old C57BL/6J mice by transection of the anteromedial meniscotibial ligament and the medial collateral ligament, and animals were euthanized 8 weeks later. Aging C57BL/6 mice were kept under normal conditions and knee joints were compared at 6, 12 and 24 months of age. Knee joints from surgical OA and aging mice groups were resected, fixed in 10% zinc-buffered formalin (Z-Fix; Anatech, Battle Creek, MI, USA) for 2 days, decalcified in TBD-2 (Shandon, Pittsburgh, PA, USA) for 72 hours, and paraffin embedded using standard protocols.

### Immunohistochemistry

Mouse knee cartilage sections were pretreated with hyaluronidase (2 mg/ml) for 1 hour before being treated with 3% (vol/vol) H_2_O_2 _for 10 minutes. The sections were then blocked with 10% goat serum for 2 hours at room temperature. After washing with Tris-buffered saline, rabbit antibodies to phospho-LKB1 (Ser428; 1:50 dilution), phospho-AMPKα (Thr172; 1:50 dilution) and the negative control rabbit IgG (1 μg/ml) were applied to the sections and incubated overnight at 4°C. Next, the sections were washed with Tris-buffered saline, incubated with biotinylated goat anti-rabbit IgG secondary antibody for 30 minutes, and then incubated for 30 minutes using the Histostain Plus kit (Invitrogen, Carlsbad, CA, USA). Finally, the sections were washed and incubated with 3,3'-diaminobenzidine substrate for 2 to 5 minutes.

### Quantification of positive staining chondrocytes

Positive staining cells in the noncalcified region of femoral and tibial cartilage of each mouse knee section were counted from six different areas of the knee (representing the center of the femoral condyle and tibia that are not covered by the menisci as well as the medial and lateral femoral condyles and tibia) adapted from the previous method [16]. The cellularity was quantified by counting the number of cells stained with hematoxylin on corresponding, adjacent sections. The number of positive cells for each antibody was expressed as the percentage of positive staining cells via immunohistochemistry, relative to the number of cells stained with hematoxylin in corresponding sections.

### Knockdown of LKB1 in human knee articular chondrocytes

Normal cultured primary human knee articular chondrocytes (passage 1) were transfected with siRNAs of LKB1 and nontarget control. Transfection used the Amaxa Nucleofection™ system (Amaxa Inc., Gaithersburg, MD, USA), according to the manufacturer's protocol. Knockdown of expression of LKB1 was examined by SDS-PAGE/western blot analysis.

### Bovine knee chondrocytes subjected to biomechanical injury

Bovine articular chondrocytes, isolated from mature cow knees as described previously [17], were embedded in 2% alginate discs with 6 mm diameter and 3 mm height. The chondrocyte-alginate constructs were then cultured in DMEM medium, containing 10% FBS and 1% penicillin-streptomycin, in a 37°C, 5% CO_2 _incubator for 3 to 4 weeks. This time period was based on prior knowledge that chondrocytes embedded in alginate require approximately 3 weeks to establish a consistent pericellular and territorial extracellular matrix [18]. This approach allowed for robust extracellular matrix production before application of biomechanical injury, which used a custom-made mechanical compression apparatus housed inside a 37°C, 5% CO_2 _incubator. Sub-lethal injury was optimized to induce no significant increase cell death at a time point immediately after completion of compression (relative to no compression), with cell viability determined *in situ *by the Live/Dead cell viability assay (Invitrogen). The sub-lethal injury condition (defined as no immediate cell death), was associated with delayed cell death (to about 25% of cells) after injury. The conditions involved continuous dynamic unconfined compression at 24% strain, with 12% amplitude at 0.5 Hz for 16 hours. The alginate-chondrocyte constructs were then cultured and analyzed at 0, 1, 2 and 5 days after injury. Chondrocytes were isolated from the constructs by dissolution of alginate using a 50 mM ethylenediamine tetraacetic acid/PBS solution, and the cell lysates were subjected to SDS-PAGE/western blot analysis for expression of cleaved caspase-3, an apoptosis marker, and phosphorylation of LKB1 and AMPKα. Conditioned media were also collected for measurement of release of nitric oxide (NO) and glycosaminoglycans (GAGs) [13]. Expression of MMP-3 was determined at both the mRNA level by quantitative RT-PCR analysis and the protein level by SDS-PAGE/western blot analysis of the conditioned media.

### Statistical analyses

All data were uniformly expressed as the mean ± standard deviation. Statistical analyses were performed by two-way analysis of variance with Bonferroni *post-hoc *test using GraphPad PRISM 5 (GraphPad, La Jolla, CA, USA). *P *<0.05 was considered significant.

## Results

### Inhibition of phosphorylation of AMPKα and enhanced catabolic responses to IL-1β and TNFα via LKB1 knockdown in chondrocytes

**Figure 1:**
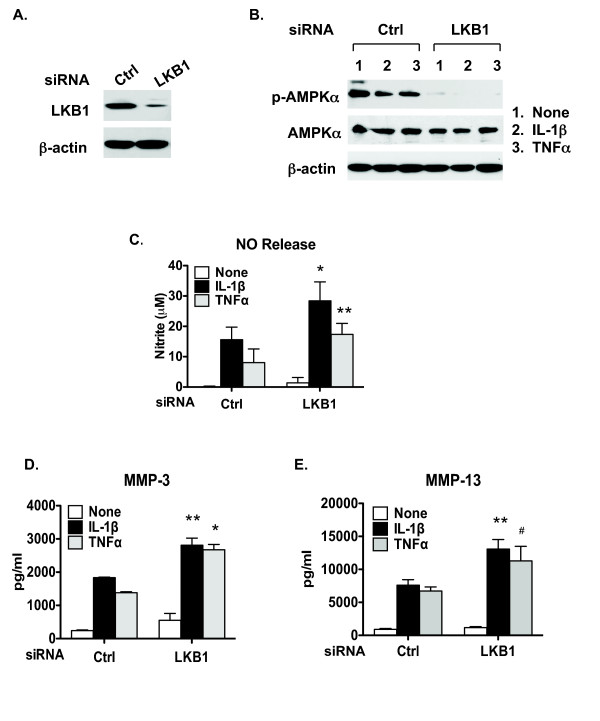
**Knockdown of LKB1 in articular chondrocytes attenuates AMPK activity and promotes matrix catabolic responses**. Human primary normal knee articular chondrocytes were transfected with liver kinase B1 (LKB1) and control siRNA. Two days after transfection, cells were either **(A) **used directly for western blot analysis of LKB1 expression or **(B) **treated with IL-1β (10 ng/ml) and TNFα (10 ng/ml) for 18 hours followed by western blot analysis for phosphorylation of AMP-activated protein kinase alpha (AMPKα) and total AMPKα. **(C) **Nitric oxide (NO) release, **(D) **matrix metalloproteinase (MMP)-3 release and **(E) **MMP-13 release were then analyzed from the conditioned media by Griess reaction and ELISA, respectively. Data representative of three individual experiments. *P <0.001, **P <0.01, #*P *<0.05 relative to the control (Ctrl).

To determine whether LKB1 regulates AMPK activity in chondrocytes, we knocked down LKB1 expression in normal cultured human knee articular chondrocytes (passage 1) via transfection with siRNAs of LKB1 and the nontarget control. The significant decrease in LKB1 protein expression by siRNA was confirmed by western blot (Figure 1A). Next, we examined phosphorylation of AMPKα in the LKB1 knockdown chondrocytes and in the control cells in the presence or absence of IL-1β and TNFα. As with our previous observation [13], phosphorylation of AMPKα was constitutively present but was decreased by IL-1β and TNFα in the control cells (Figure 1B). In comparison, phosphorylation of AMPKα was almost completely inhibited at the basal level and was blunted by IL-1β and TNFα in the LKB1 knockdown chondrocytes (Figure 1B), indicative of a necessary regulatory role of LKB1 for AMPK activation in chondrocytes. In these experiments, we saw increased NO release by 82% and 115% in response to IL-1β and TNFα, respectively, compared with control cells (Figure 1C). In addition, release of MMP-3 and MMP-13 in response to IL-1β and TNFα were also enhanced in LKB1 knockdown chondrocytes (Figure 1D,E). Conversely, overexpression of LKB1 in chondrocytes via transfection prevented dephosphorylation of AMPKα and inhibited NO production and MMP-3 and MMP-13 release in response to IL-1β and TNFα (data not shown).

### Loss of phosphorylation of LKB1 and AMPKα in cultured human knee osteoarthritic chondrocytes

**Figure 2:**
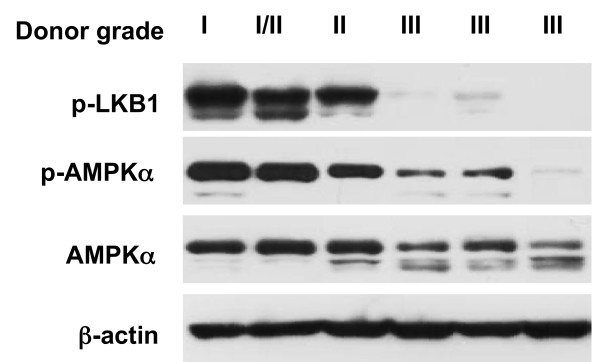
**Loss of phosphorylation of both LKB1 and AMPKα in human knee osteoarthritic chondrocytes**. Lysates of cultured primary human knee chondrocytes (grade I to III) were subjected to western blot analysis with antibodies to phospho-liver kinase B1 (p-LKB1; Ser428) and phospho-AMP-activated protein kinase alpha (p-AMPKα; Thr172), as described in Materials and methods. Data representative of three individual experiments (total of 15 different donors).

Since decreased phosphorylation of AMPKα was previously observed in human knee OA chondrocytes [13], we tested here for concurrent decrease of chondrocyte LKB1 and AMPKα phosphorylation. Cultured primary human chondrocytes (passage 1) with a range of normal (grade I), mild OA (grade II) and OA (grade III) were subjected to SDS-PAGE/western blot analysis of phosphorylation of LKB1 and AMPKα. Phosphorylation of LKB1 was constitutively present in grade I and grade II chondrocytes, but was substantially decreased in grade III chondrocytes (Figure 2), where there was decrease in AMPK phosphorylation (Figure 2).

### Concomitant reduction of phosphorylation of LKB1 and AMPKα in mouse knee osteoarthritic and aged mouse knee cartilages *in situ*

**Figure 3:**
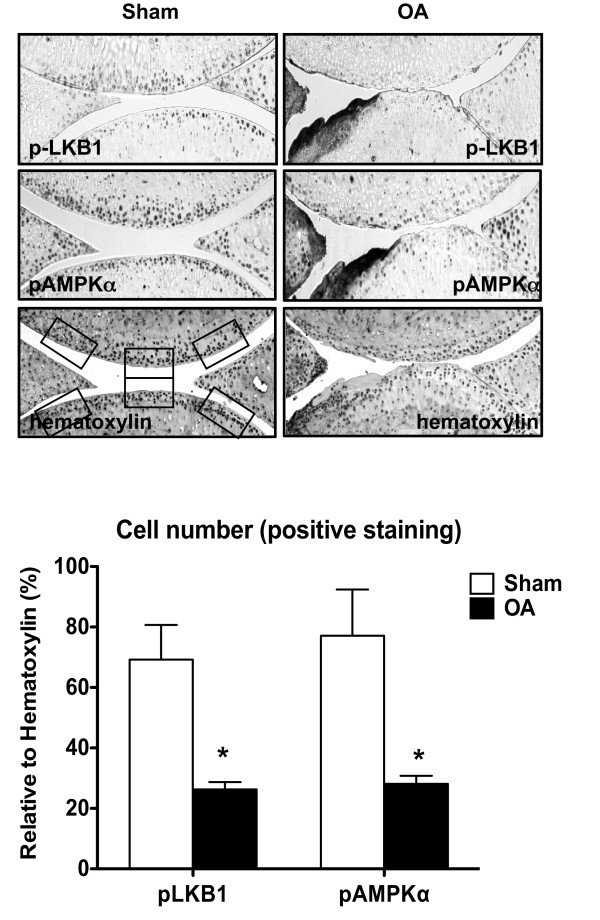
**Concomitant reduction of phosphorylation of LKB1 and AMPKα in mouse knee osteoarthritic cartilages**. Mouse knee sections from surgical instability-induced osteoarthritis (OA) and the sham control at 8 weeks post surgery were analyzed for phosphorylation of liver kinase B1 (LKB1) and AMP-activated protein kinase alpha (AMPKα) by immunohistochemistry as described in Materials and methods. Cellularity of each section was confirmed with hematoxylin staining. Only the cells present in the noncalcified region of femoral and tibial cartilage were subjected to analysis. Cells staining positively for LKB1 and AMPKα were presented as percentage of cells stained for hematoxylin. Data representative of two individual experiments (*n *= 3 animals). *P <0.05 relative to the sham control.

**Figure 4:**
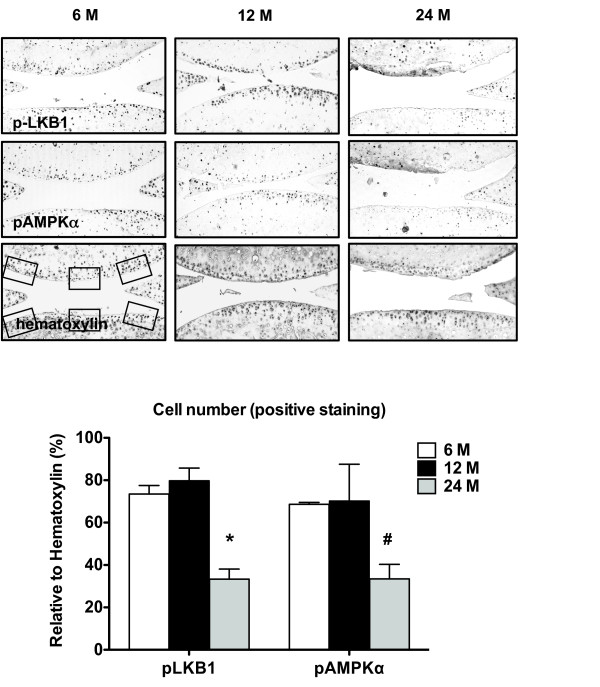
**Concomitant reduction of phosphorylation of LKB1 and AMPKα in aged mouse knee cartilages**. Knee sections of mice 6, 12 and 24 months old were analyzed for phosphorylation of liver kinase B1 (LKB1) and AMP-activated protein kinase alpha (AMPKα) by immunohistochemistry, as described in Materials and methods. Cellularity of each section was confirmed with hematoxylin staining. Only the cells present in the noncalcified region of femoral and tibial cartilage were subjected to analysis. Cells staining positively for LKB1 and AMPKα were presented as percentage relative to cells stained for hematoxylin. Data representative of two individual experiments (*n *= 4 for each time point). *P <0.01, #*P *<0.05 relative to the 6-month-old mouse knee cartilage.

Immunohistochemistry analysis of mouse knee sections of surgically induced OA and sham control (with Osteoarthritis Research Society International scores of 5 and 0, respectively) revealed that phosphorylated LKB1 and AMPKα were concomitantly decreased in mouse OA knee cartilage, compared with the sham control (Figure 3). Additionally, immunohistochemistry analysis of mouse knee sections 6, 12 and 24 months old (with Osteoarthritis Research Society International scores of 0.5, 1 and 1, respectively) revealed that the numbers of cells staining positively for either phosphorylated LKB1 and AMPKα were similar in 6-month-old and 12-month-old mouse knee cartilages, but were concomitantly decreased in 24-month-old mice knee cartilages (Figure 4).

### Decreased phosphorylation of LKB1 and AMPKα and increased apoptosis and catabolic responses in chondrocytes after biomechanical injury *in vitro*

**Figure 5:**
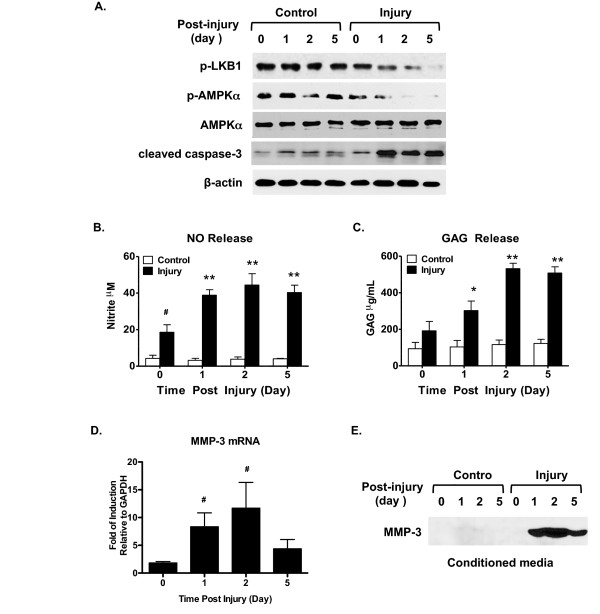
**Decreased LKB1 and AMPKα phosphorylation and increased apoptosis and catabolic responses after biomechanical injury**. Loss of phosphorylation of liver kinase B1 (LKB1) and AMP-activated protein kinase alpha (AMPKα), in addition to increased caspase-3 activation, nitric oxide (NO) release, and catabolic activities in bovine chondrocytes after biomechanical injury. Bovine knee articular chondrocytes were embedded in alginate disks and cultured for 3 to 4 weeks, followed by the sub-lethal biomechanical injury condition described in Materials and methods. The chondrocyte-alginate constructs were collected at 0, 1, 2 and 5 days after injury. **(A) **Chondrocytes from both injured and control groups were then released by dissolution of alginate, and subjected to western blot analysis for expression of cleaved caspase-3, phosphorylation of LKB1 and AMPKα, and total AMPKα. Conditioned media were employed for analysis of **(B) **NO and **(C) **glucosaminoglycan (GAG) release by Griess reaction and DMMB assay, respectively, and **(E) **matrix metalloproteinase (MMP)-3 release by western blot analysis. **(D) **MMP-3 mRNA expression was also examined by quantitative RT-PCR. Data representative of three individual experiments (*n *= 3 replicates for each condition). **P <0.001, *P <0.01, #*P *<0.05 relative to the control.

We cultured normal bovine knee chondrocytes embedded in 2% alginate (three-dimensional) for 3 to 4 weeks to allow extracellular matrix production [18] before subjecting them to sub-lethal injury by continuous dynamic unconfined compression at 24% strain, 12% amplitude, 0.5 Hz for 16 hours as described in Materials and methods. The chondrocyte-alginate constructs were then cultured and collected at 0, 1, 2 and 5 days after injury. As seen in Figure 5A, activation of caspase-3, an apoptosis mediator, became detectable in chondrocytes at 1, 2 and 5 days after compression. In contrast, phosphorylation of LKB1 and AMPKα started to decrease at 1 day and became attenuated at 5 days post injury (Figure 5A). Significant induction of NO release was seen immediately after injury and leveled off at 2 days post injury (Figure 5B). Similar results were observed for GAG release (Figure 5C) that could reflect matrix catabolism. Induction of MMP-3 expression was seen at both mRNA and protein levels, with the highest level at 2 days after compression (Figure 5D,E). Taken together, decreased concurrent phosphorylation of LKB1 and AMPKα were associated with increased proapoptotic and catabolic responses in chondrocytes following mechanical injury.

### Suppression of increased catabolic responses to injury by AMPK pharmacologic activators in cultured chondrocytes

**Figure 6:**
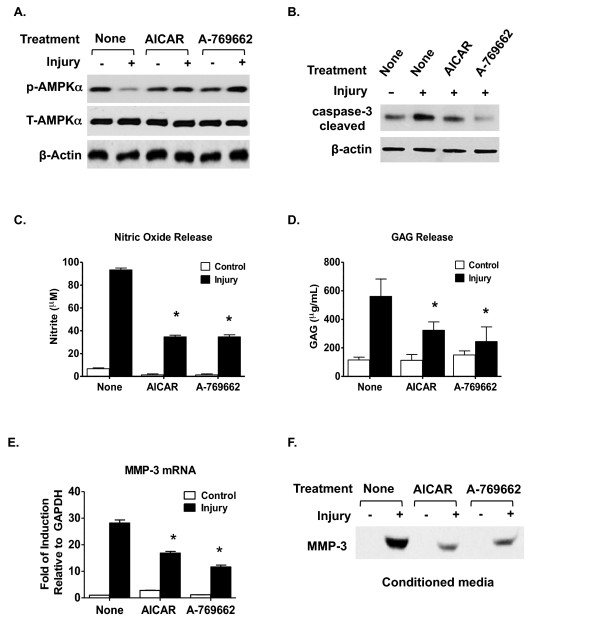
**AMPK pharmacologic activators inhibit catabolic responses of chondrocytes to biomechanical injury**. Bovine chondrocyte-alginate disks were pretreated with 5-aminoimidazole-4-carboxyamide ribonucleoside (AICAR; 1 mM) and A-769662 (0.5 mM) for 24 hours, and then subjected to mechanical injury as described in Materials and methods. After culturing for 2 days, the chondrocytes released from alginate and the conditioned media were used for analysis of **(A) **phosphorylation of AMP-activated protein kinase alpha (AMPKα) and total AMPKα, **(B) **cleaved caspase-3, **(C) **nitric oxide release and **(D) **glucosaminoglycan (GAG) release, and **(E, F) **matrix metalloproteinase (MMP)-3 expression. Data representative of two individual experiments (*n *= 3 replicates for each condition). *P <0.01 relative to the control.

Bovine knee chondrocytes embedded in alginate, which had first been cultured for 3 to 4 weeks, were pretreated with AMPK pharmacologic activators AICAR (1 mM) and A-769662 (0.5 mM) for 24 hours before being subjected to biomechanical injury. After culture for 2 days post injury, the chondrocyte-alginate constructs and the conditioned media were collected for analysis of catabolic responses. As shown in Figure 6, both AICAR and A-769662 prevented dephosphorylation of AMPKα (Figure 6A) and inhibited caspase-3 activation (Figure 6B) after mechanical injury. In parallel, both AICAR and A-769662 dramatically inhibited NO and GAG release and MMP-3 expression at both mRNA and protein levels after mechanical injury (Figure 6C,D,E,F).

## Discussion

This study demonstrated that activation of AMPK requires LKB1 in chondrocytes. Knockdown of LKB1 expression via siRNA resulted in attenuation of basal phosphorylation of AMPKα, as well as significant enhancement of catabolic responses to IL-1β and TNFα in chondrocytes. Conversely, overexpression of LKB1 in chondrocytes via transfection prevented dephosphorylation of AMPKα and inhibited NO production and MMP-3 and MMP-13 release in response to IL-1β and TNFα (data not shown). We observed concomitant loss of phosphorylation of LKB1 and AMPKα in human knee OA chondrocytes (grade III) and in mouse knee OA cartilages.

Aging is a major risk factor for development of OA [1-4]. Decreased AMPK activation is linked with several age-associated diseases [19]. For example, deficiency of AMPK exacerbates aging-induced myocardial dysfunction [20]. Aging also impairs AMPK activation and suppresses insulin-stimulated glucose uptake into rat skeletal muscles, which is held to enhance the development of metabolic syndrome [21]. Here, we observed that phosphorylation of both LKB1 and AMPKα were present at 6 and 12 months of age in mouse knee cartilages, but were reduced significantly in 24-month-old mouse knee cartilages. Our results suggest that decreased capacity for linked LKB1 and AMPK activation in aging chondrocytes may increase susceptibility to OA development.

Biomechanical injury can promote the development and progression of OA [7,22]. Our translational studies of the pharmacologic AMPK activators AICAR and highly AMPK-selective A-769662 suggested that maintaining AMPK activity in injured chondrocytes can promote preservation of cartilage matrix integrity. Significantly, we established and validated a new, sub-lethal biomechanical injury condition for chondrocytes in three-dimensional chondrocyte-alginate constructs. In this system, concurrent loss of phosphorylation of LKB1 and AMPKα, associated with induction of caspase-3 activation, release of NO and GAG, and MMP-3 expression, developed in response to injury. Conversely, treatment of chondrocyte-alginate constructs with AMPK pharmacologic activators AICAR and A-769662 prevented dephosphorylation of AMPKα and inhibited caspase-3 activation and catabolic responses induced by biomechanical injury.

There are several core limitations to this study. First, chondrocytes can retain their phenotype and produce physiological cartilage matrix components when immobilized in alginate, but the biomechanical properties of three-dimensional chondrocyte-alginate constructs may not be identical to those in articular cartilage. Future biomechanical injury studies with articular cartilage explants would be of interest. Second, the scope of analyses herein focused on the relationship between LKB1 and AMPK activity. However, regulation of AMPK involves phosphorylation by two upstream kinases in addition to LKB1; that is, calcium calmodulin-dependent kinase kinase beta and transforming growth factor beta-activated kinase 1 [10,11]. Whether calcium calmodulin-dependent kinase kinase beta and transforming growth factor beta-activated kinase 1 regulate AMPK activation in chondrocytes remains to be determined. Protein phosphatases such as protein phosphatase 2A and protein phosphatase 2C negatively regulate AMPK activity through dephosphorylation of AMPKα [10,11]. Unlike the case for LKB1, however, we have not observed any significant differences in expression of protein phosphatase 2Cα between human knee OA (grade III) and normal chondrocytes (grade I) (R. Liu-Bryan, unpublished observation). Third, AMPK promotes autophagy [23,24], which may be a protective mechanism against injury in chondrocytes that decreases in aging cartilage [16,25]. Analysis of the relationships between LKB1 and AMPK activity and autophagy in aging cartilage and biomechanically injured chondrocytes were beyond the scope of this study, but would be of interest.

## Conclusion

Reduction in functionally linked LKB1 and AMPK activation in chondrocytes associated with aging, inflammation and biomechanical injury has the potential to disrupt cartilage extracellular matrix homeostasis, and thereby could contribute to OA development and progression. Pharmacologic AMPK activation is a novel potential therapeutic approach for OA that merits further investigation.

## Abbreviations

AICAR: 5-aminoimidazole-4-carboxyamide ribonucleoside; AMPK: AMP-activated protein kinase; DMEM: Dulbecco's modified Eagle's medium; ELISA: enzyme-linked immunosorbent assay; FCS: fetal calf serum; GAG: glucosaminoglycan; IL: interleukin; LKB1: liver kinase B1; MMP: matrix metalloproteinase; NO: nitric oxide; NF: nuclear factor; OA: osteoarthritis; PBS: phosphate-buffered saline; PCR: polymerase chain reaction; RT: reverse transcriptase; TNF: tumor necrosis factor.

## Competing interests

RL-B who holds a patent (filed through University of California, San Diego, CA, USA) that partially related to the content of the manuscript; however, RL-B has not received any reimbursements, fees, funding, or salary from this organization that holds the patent relating to the content of the manuscript. The remaining authors declare that they have no competing interests.

## Authors' contributions

RL-B and RT had full access to all data in the study and take responsibility for the integrity of the data and accuracy of the data analysis. FP and RL-B were responsible for acquisition of data. FP, MH, RJ, ML, RT, and RL-B were responsible for study conception and design, and for analysis and interpretation of data. All authors were involved in drafting or revising the manuscript critically for important intellectual content, and all authors approved the final version to be published.
